# Supplementary material for: Are carbon emissions trading and green financial instruments synergistic? -Comprehensive quantitative research based on content analysis
Source: PLoS One. 2024 Mar 7;19(3):e0298601. doi: 10.1371/journal.pone.0298601 (PMC10919601; doi:10.1371/journal.pone.0298601)
Supplement: S1 Data — (ZIP) [file pone.0298601.s001.zip › Data/地级市Z_F工作报告.zip!/╦─┤¿/╤┼░▓╩╨/└┤╫╘╣1⁄2╓┌║┼í╛┬φ┐╦╩2╛▌═°í┐_2016.docx]

各位代表：

现在，我代表市人民政府，向大会作政府工作报告，请予审查，并请市政协委员提出意见。

2015年和“十二五”工作回顾

2015年是灾后恢复重建的攻坚之年，也是全面完成“十二五”规划的收官之年。在市委的坚强领导下，在市人大、市政协的监督支持下，市人民政府认真贯彻落实党中央、国务院，省委、省政府和市委的各项决策部署，坚持重建脱贫“双攻坚”，大力实施后发追赶，全面完成了市三届人大五次会议确定的各项目标任务。纳入灾后恢复重建总体规划的2160个项目累计完工1949个，完工率90.2%；累计完成投资646.3亿元，投资完成率90.6%。实现地区生产总值502.58亿元，增长9%；地方一般公共预算收入30.31亿元，增长10.7%；城镇居民人均可支配收入25318元，增长8.1%；农村居民人均可支配收入10195元，增长9.9%。

一年来，我们主要做了以下工作。

凝心聚力实施重建攻坚。把牢重建中心，严格执行重建规划，坚持目标倒逼、任务倒逼、责任倒逼，下深水、破难题、克难关，圆满完成了“双90%”的年度目标任务，灾后恢复重建取得阶段性重大成效。突出保障和改善民生，城乡居民住房和公共服务设施重建优先推进。全面完成33696套城房重建任务并同步做好和谐分房工作，新村聚居点配套基础设施逐步完善，“自管委”管理服务职能不断健全。加快推进公共服务设施重建，370所基础教育学校（含幼教）于秋季开学前全部完工并投入使用，教育和医疗卫生类重建项目完工率分别比全市重建平均进度高8.8个和5.12个百分点。陆家坝体育活动中心基本建成，市卫计服务中心主体工程完工，市文化中心即将竣工。围绕提升灾区发展条件和城镇化水平，基础设施和城镇体系重建顺利推进。坚持把恢复功能与提升水平有机结合，国道108线改造、国道351线多功至芦山县城段建设全面完成，国道318线改造主体工程完工，芦山生态环线基本建成，永兴大道实现通车。科学实施城镇体系重建，中心城区、6个县城和16个重点镇重建工作顺利推进。水利、能源、通信等重建项目加快实施，名山农村供水总厂建成运行，有效解决了近24万人安全饮水问题。着眼灾区长远发展和群众致富奔康，产业重建加快推进。着力强化产业园区建设，一批新的增长点加快形成。中恒天汽车雅安项目获得整车生产资质，新筑通工新能源公交汽车项目正式投产，建安车桥项目基本建成，飞地经济发展模式得到省委、省政府充分肯定。坚持“一三互动、农旅结合”，初步建成3条百公里百万亩特色产业走廊和5个农业示范园。生态环境加快修复，地质灾害防治工作扎实有效，地震灾区连续三年实现地质灾害零伤亡。对口援建工作进展顺利，对口合作不断深化拓展。按照“出合格成品、建优质精品、见重建作品”的要求，统筹“点、线、片、面”提升重建水平。坚持和深化“五总”〔注1〕推进机制，制定实施重建水平整体提升方案，突出抓好“一城四镇”〔注2〕“两道一路”〔注3〕“四个园区”〔注4〕等重点区域、重要线路和重大项目的示范带动，重建水平实现整体提升。坚持问题导向，强化风险防控，工作底线全程全面坚守。成立“六位一体”工作底线〔注5〕监督检查工作领导小组，建立完善定期报告、社会评议等制度，加强对安全、质量和资金使用等环节的全程监管，在省审计厅支持下大力开展对口援助审计，持续深入开展“回头看”、拉网式检查，及时将问题整改落实到位，确保科学重建、阳光重建、廉洁重建。

主动作为稳定经济增长。始终保持专注发展定力，努力克服经济下行压力，经济呈现稳中向好发展态势。强化政策激励稳增长。在全面落实中央、省系列稳增长政策措施的基础上，针对我市经济发展面临的突出困难和问题，从财政扶持、电力支持、企业融资、市场拓展等方面制定了12条具体措施。用好市级工业发展资金、中小企业应急转贷资金，抓好“助保贷”“园保贷”和“票据贴现补助”，实施留存电量政策和临时电价干预等措施，大力支持实体经济发展。狠抓项目投资稳增长。坚持经济工作项目化、项目工作责任化，切实抓好项目储备、开工和建设，着力实施了一批夯基础、增后劲、惠民生的重大项目。川藏铁路成都（朝阳湖）至雅安段、雅康高速公路加快建设；锅浪跷水电站全面开工，大岗山水电站投产发电；铜头引水、九龙水库、南郊水厂引水工程顺利推进，永定桥水利工程正式向汉源县城供水。完成全社会固定资产投资531.41亿元，增长12.7%。激活市场消费稳增长。积极组织参加市场拓展“三大活动”〔注6〕，着力搭建消费平台，不断扩大市场消费，大力培育新的消费热点，社会消费品零售总额增长12.6%。鼓励创业创新稳增长。实施创新驱动发展战略，全面推进大众创业、万众创新，建成各类孵化器20家，新增高新技术企业7户、科技型中小微企业18户，大学生就业促进和创业引领计划有序实施，在外雅商和农民工、农民企业家返乡创业工作初见成效。支持县域经济发展稳增长。积极发挥特色优势，提升县域经济发展水平。6个县的地区生产总值、地方一般公共预算收入、全社会固定资产投资分别增长9.5%、11.8%和8.5%。天全县稳增长工作获国务院督查表扬，石棉县、天全县被省委、省政府通报表扬为县域经济发展先进县，名山区、石棉县分别被评为全省“三农”工作先进区、全省农民增收工作先进县，汉源县农业农村工作得到省上充分肯定。

突出特色加快产业发展。坚持扩增量、提质量，以优势资源转化带动产业转型升级。坚持工业挑大梁，工业经济提质增效。规模以上工业增加值增长10.2%。四大骨干产业产值达470.71亿元，六大特色产业产值达154.22亿元。新增规模以上企业27户，4户企业进入四川制造企业100强，4户企业在“新三板”挂牌融资。夯实农业强底座，特色农业稳步发展。第一产业增加值增长3.9%。成功创建首个全国休闲农业与乡村旅游示范市（地级市），“五雅”〔注7〕特色优势产业持续壮大，新发展产业基地40万亩，新增绿色（有机）认证面积23.5万亩。新培育市级龙头企业18户、农民专合组织337家，龙头企业销售收入增长10.7%。突出三产当尖兵，第三产业加快发展。第三产业增加值增长8.9%，占地区生产总值比重提高1.2个百分点。全面推动国家生态文化旅游融合发展试验区建设，碧峰峡创建国家5A级旅游景区顺利通过景观资源评估，云峰山、硗碛藏寨－神木垒、花海果乡、周公山、龙门古镇、喇叭河成功创建国家4A级旅游景区，神木垒景区入选国家生态旅游示范区，飞仙湖被批准为国家水利风景区。成功举办第十一届蒙顶山国际茶文化旅游节等活动，全年实现旅游综合收入152.3亿元，增长40.3%。雅安市物流园区控制性详规通过评审，川滇藏商贸物流园区基础设施建设基本完成。康养、金融、电子商务等新兴先导型服务业加快发展。

抢抓机遇深化改革开放。蹄疾步稳深化改革。扎实推进行政审批制度改革，全面取消非行政许可审批，编制完成权力清单并向社会公布；全面实施“三证合一”登记制度〔注8〕，新登记企业2519户，增长19.5%。稳步推进农村产权制度改革，农村土地承包经营权确权颁证工作完成60%。着力推进金融改革，雅安农村商业银行改革和雅安市商业银行首轮增资扩股工作有序推进。继续深化财税体制、国资国企、医药卫生体制、教育领域和文化体制等各项改革，不动产登记职能整合工作全面完成。深入拓展开放合作。主动融入“1+7”同城化发展〔注9〕，与成都市、乐山市、眉山市、甘孜州、阿坝州、凉山州、省交通运输厅和省铁投集团、省能投集团、省交投集团、西南建筑设计院等开展“十三五”战略合作，共同推动了一批交通、产业、生态等合作项目，环雅安全方位、多层次开放合作的发展新格局基本形成。大力开展招商引资。组织参加中外知名企业四川行、首届中国西部（四川）进口展暨国际投资大会等重大投资促进活动，多形式、多渠道强化招商引资工作，成功引进50亿元战略投资控股碧峰峡进行升级打造，投资50亿元的牛背山项目和30亿元的龙苍沟项目进入实质性开发，投资19亿元的国盛·雅安国际商贸城和投资15亿元的雅安万达商业广场项目落地建设。政府与社会资本合作（PPP）项目工作有序推进。全年引进到位市外资金329亿元，增长13.4%。

建管并重提升城乡面貌。着力完善城乡规划。完成大兴新区控制性详规和荥经、汉源、石棉县城总规编制，城市（含县城）总规覆盖率达100%，乡镇规划覆盖率达76.3%。加快推进城乡建设。中心城区“一城两线三出口”〔注10〕等重点区域和重要节点改造全面推进，姚桥新城功能不断完善，雅州大道、熊猫大道沿线绿化、亮化工程全面竣工，北外环线实现全线通车，成雅高速公路北郊、多营进出口大道改造和雅乐高速公路光华山隧道连接线建设全面完成。大兴大桥建成通车，大兴新区大道一段顺利完工。雨城区、名山区和芦天宝飞地产业园区（雅安经开区）同城化步伐进一步加快。6个县城综合承载能力进一步增强，7个“百镇建设行动”试点镇加快建设，“三百示范工程”〔注11〕有力有效推进并达到示范效果。切实强化城乡管理。大力推进“两城”创建〔注12〕，深入开展城乡环境综合治理，重点整治康藏路、挺进路、协和广场、青衣江公园、北外环沿线环境秩序，依法拆除违法建筑23万平方米，城市“五乱”〔注13〕现象得到有效遏制，全市乡镇、村庄（社区）农村生活垃圾治理达到“五有”〔注14〕标准。

持续强化生态文明建设。发挥雅安生态优势，扎实推进国家首批生态文明先行示范区建设。持续实施天然林资源保护、新一轮退耕还林、野生动植物保护及自然保护区建设工程，森林覆盖率达63.1%，保持全省第一。加强空气质量监测，强化大气污染联防联控，全年空气质量优良率达94%，空气中可吸入颗粒物平均浓度低于省定标准。抓好水污染治理和饮用水源保护，主城区“一江三河”〔注15〕治理全面完成，14个城市集中式饮用水源地水质100%达标。强化水土保持，治理水土流失面积205平方公里。加强资源循环利用，一名微晶被工信部和安监总局列为尾矿综合利用示范工程。淘汰12户企业落后产能，万元地区生产总值能耗和万元工业增加值能耗下降完成省定目标。

尽心竭力改善人民生活。按照“四年集中攻坚、两年巩固提升”的工作目标，制定“3+13”政策组合〔注16〕，落实“六个精准”〔注17〕要求，实施“五个一批”〔注18〕行动计划，扎实推进九项重点扶贫工作〔注19〕，精准扶持52104名农村扶贫对象改善生产生活条件，脱贫攻坚实现良好开局。全面完成“十项民生工程”和“20件民生实事”，民生支出占一般公共预算支出比重达66%。切实抓好就业促进和社会保障工作，城镇新增就业20856人，城镇登记失业率3.81%；城乡居民基本养老保险和城镇职工养老保险分别覆盖41.13万人、34.76万人，新农合参合率达99.69%，城镇居民医保参保率达98%，“五保”〔注20〕对象集中供养率达62%，城乡居民月人均最低生活保障标准分别提高70元和55元。开工建设保障性住房和实施危旧房棚户区改造3540套，竣工3703套。落实教育惠民政策，“三免一补”工程〔注21〕扶助学生27.29万人次，资助普通高中和中职教育家庭经济困难学生5.08万人次。8县（区）义务教育均衡发展通过省政府督导评估，高考本科上线突破3000人，雅职院成功创建省级示范性高职院校。健全市、县、乡、村四级医疗卫生服务体系，深化县级公立医院综合改革，推行分级诊疗制度，数字化医院建设全面完成。完成市广播电视台和雨城电视频道资源整合，组织承办国家级、省级全民健身活动10次。切实做好妇女、儿童、老龄、残疾人、社会福利、慈善、红十字工作，防震减灾、气象、档案、保密、科协、地方志、社会科学工作扎实推进，外事、侨务和对台等工作取得新进展。

依法治理维护社会稳定。大力推进法治雅安、平安雅安建设，“六五”普法工作通过省级验收。加强基层政权建设，创新社会治理体制和群众工作方法。强化社会治安综合治理，健全社会稳定风险评估和社情民意调查机制，深入开展网格化服务管理，持续推进“法律进重建、调解进项目”工作，矛盾纠纷化解成功率达95.1%。深化反分裂、反渗透、反恐怖斗争，持续保持对各类违法犯罪的严打高压态势，社会大局保持稳定，政风行风群众安全感、满意度测评综合指数排名全省第二。加强重点行业和领域安全专项整治，科学有效处置石棉“9·26”液苯罐车交通事故，全年未发生重特大安全生产事故。加强食品药品安全监管，专业、高效的食品药品安全监管体系基本形成。国家安全、民族、宗教工作有序推进，国防动员和民兵预备役工作取得新成效。

从严从实加强自身建设。扎实开展“三严三实”专题教育，政府执行力、公信力和依法行政能力得到有效提升。自觉接受市人大及其常委会的法律监督、工作监督和市政协的民主监督，认真办理人大代表建议意见151件、政协委员提案154件，办结率100%。认真听取民主党派、工商联、无党派人士和人民团体的意见，虚心接受群众监督和社会舆论监督。健全依法决策机制，完善绩效管理体系，深化“止懒治庸”升级版。落实中央“八项规定”精神，严格执行省委省政府“十项规定”，坚决防止“四风”〔注22〕反弹，市级“三公经费”〔注23〕下降2.1%。深入推进反腐倡廉建设，严肃查处了一批违纪违法案件。

各位代表，总结2015年工作，我们深感欣慰和自豪；回顾“十二五”，我们更感不平凡、不寻常。“十二五”时期，是雅安发展历史上极为特殊、极其艰难的五年。五年来，面对多种任务聚集、多重压力叠加、多条战线作战的复杂局面，我们坚持稳中求进总基调，积极有效应对各种困难挑战，统筹推进稳增长、促改革、调结构、惠民生、防风险各项工作，实现了在大灾后奋力崛起、在逆境中后发追赶，经济社会发展取得了令人鼓舞的巨大成就。

五年来，我们战胜灾难、坚韧奋起，认真实施了重建新路的雅安实践。在胜利完成“5·12”汶川特大地震灾后重建任务后，我市又遭受了“4·20”芦山强烈地震。在党中央、国务院的亲切关怀和省委、省政府的坚强领导下，全市上下万众一心、众志成城，打赢了抢险救灾、安置群众、抢通保通、卫生防疫、防治次生灾害等一场场硬仗，最大限度地挽救了群众生命，最大限度地降低了灾害损失。进入灾后重建后，我们认真贯彻落实习近平总书记、李克强总理等中央领导以及省委、省政府领导的重要讲话、指示和批示精神，按照“三年基本完成、五年整体跨越、七年同步小康”的目标要求，坚持以城乡住房和公共服务设施重建推动社会民生改善，民生事业实现跨越发展；以城镇体系和基础设施重建促进城乡整体水平提升，城乡面貌发生巨大变化；以产业重建带动三次产业发展，自我造血功能得到极大提升；以防灾减灾体系建设和生态修复为重点，抵御和应对重大自然灾害的能力显著增强；以坚守“六位一体”工作底线保障了重建工作经得起组织、群众和历史的检验。经过两年多“灾后恢复重建新路子”的雅安实践，灾区已经走出地震阴霾，正在发生脱胎换骨的巨大变化，“户户安居有业、民生保障提升、产业创新发展、生态文明进步、同步奔康致富”的规划愿景正在变为看得见、摸得着的生动现实。

五年来，我们化危为机、主动作为，奋力开创了后发追赶的崭新局面。面对持续加大的经济下行压力和艰巨繁重的灾后重建任务，我们化压力为动力，变挑战为机遇，按照市委三届七次全会确立的“抓住灾后恢复重建大机遇，做好生态文明示范大文章，加快建设幸福美丽新家园”的总体思路，坚持在打重建“硬仗”中补发展“欠账”，坚定不移地走后发追赶之路。“十二五”期间，全市地区生产总值迈上500亿元台阶，是“十一五”末的1.75倍，年均增长10.6%；人均地区生产总值、地方一般公共预算收入、社会消费品零售总额年均分别增长11.5%、14.1%和14.2%；全社会固定资产投资累计完成2053.8亿元，是“十一五”时期的1.77倍；三次产业结构由“十一五”末的17.4∶55.1∶27.5调整为14.4∶55.9∶29.7。全市经济实现了量的扩张和质的提升，正迈步踏上发展振兴、追赶跨越的新征程。

五年来，我们抢抓机遇、前瞻布局，着力奠定了长远发展的坚实基础。抢抓灾后恢复重建和国家实施“一带一路”〔注24〕、长江经济带建设以及新一轮西部大开发等机遇，着力加快基础设施和生态环境建设，雅安发展条件进一步改善，后发优势更加凸显。“十二五”期间，新建高速公路189公里，新改建国省干道897公里、农村公路2200公里，川藏铁路成都（朝阳湖）至雅安段开工建设，雅西、雅乐、邛名高速公路建成通车。全市城镇化率达42.55%，比“十一五”末提高7.95个百分点；完成名山撤县设区工作，中心城区建成区面积达31.21平方公里，比“十一五”末增加10.21平方公里。全市水电总装机容量达1256万千瓦，比“十一五”末增加349万千瓦。雅安成功纳入国家首批生态文明先行示范区、国家生态文明示范工程试点市和国家生态文化旅游融合发展试验区，生态文明建设迈出重要步伐。

五年来，我们以人为本、发展为民，努力实现了民生福祉的持续改善。顺应人民群众对幸福美好生活的新期待，以更大力度、更高标准办成了一批既利当前、又惠长远的民生实事。“十二五”期间，一般公共预算民生投入达629.97亿元，是“十一五”时期的4.67倍。城乡居民人均可支配收入年均分别增长12.1%和13.9%。城镇新增就业9.52万人，解决87万农村居民安全饮水问题，改造各类棚户区和城市农村危房10.6万套。扶持20.69万相对贫困人口改善生产生活条件，绝对贫困发生率从9.1%下降到2.56%。科技、教育、卫生、文化、体育等各项社会事业全面发展。“瀑电”移民工作全面完成，库区群众生产生活进一步改善。

成绩来之不易，这是党中央、国务院，省委、省政府坚强领导、亲切关怀的结果，是市委统揽全局、科学决策的结果，是市人大、市政协履职监督、共同努力的结果，是省级部门、兄弟市（州）和社会各界关心帮助、倾力支持的结果，是全市干部群众齐心协力、共同奋斗的结果。在此，我谨代表市人民政府，向全市人民，向所有关心、支持和参与雅安建设发展的各界人士，表示衷心的感谢，并致以崇高的敬意！

回顾五年来的工作，有七个方面的经验和体会特别值得总结，并在今后的工作中继续加以坚持：不断强化不发展要落后、发展慢了也要落后的忧患意识，是推进雅安后发追赶的强大内在动力；敢于担当、超常付出，是化危为机、崛起于危难的根本举措；坚持转化优势、特色发展，是适应经济发展新常态、顺应全国全省发展大势的科学抉择；坚持问题导向、目标引领、责任倒逼，是遵循发展规律、破解发展难题的有效做法；坚持发展为民、重建惠民、生态富民，是践行为民宗旨的具体体现；坚守工作底线、强化风险防控，是牢牢把握主动权、让发展经得起历史检验的重要基础；坚持依法行政、从严治政，在思想上、政治上、行动上与党中央、省委和市委保持高度一致，坚决贯彻落实市委决策部署，持之以恒地转作风、抓落实，是保持正确前进方向、加快各项事业发展的坚强保证。

在看到成绩的同时，我们也清醒地认识到：灾后重建中还存在部分项目进度不平衡和管理不到位等问题，重建水平有待进一步提升；全市经济总量偏小，工业质量效益不高，服务业占比偏低，生态、区位等比较优势没有得到充分转化，县域经济不强的现状没有得到根本性转变；后续项目储备不足，投资快速增长难度较大；部分县（区）、部门抓招商引资的积极性和主动性没有充分激发，投资发展环境有待进一步优化；城镇化进程滞后于全国、全省平均水平，城乡规划、建设和管理水平有待进一步提升；脱贫攻坚任务依然繁重，教育、医疗、养老等公共服务与群众期待还有较大差距，城乡群众生产生活中还有不少突出问题亟待解决；部分干部思想观念不够解放，政府自身建设有待进一步加强。这些问题，我们将高度重视，并在今后的工作中切实加以改进。

“十三五”发展的基本思路

“十三五”时期是全面建成小康社会的决胜阶段，也是我市实施追赶跨越的战略机遇期。根据市委关于“十三五”规划编制的建议，市人民政府组织编制了《雅安市国民经济和社会发展第十三个五年规划纲要（草案）》（以下简称《规划纲要》），已印发大会审议。这里，我着重从四个方面简要说明。

关于发展形势。《规划纲要》的编制，深刻把握了全国、全省发展形势和我市的阶段性特征。今后五年，我国仍处于可以大有作为的重要战略机遇期，经济长期向好的基本面没有改变，但内涵已发生深刻变化，经济风险发生概率上升，发展面临诸多严峻挑战。全省经济发展已进入规模质量同步提升期，正在积蓄新的发展动能，同时又将经历转型阵痛，面临严峻挑战。经过近三年重建，我市的发展基础和发展能力得到了显著提升，但底子薄、总量小、不平衡等仍然是最突出的短板。为此，我们必须进一步强化忧患意识、机遇意识和责任意识，把继续打“硬仗”、补“欠账”作为贯穿“十三五”期间的总要求，保持定力、乘势而进，加快追赶跨越步伐，确保与全国、全省同步全面建成小康社会。

关于发展理念。“十三五”时期，必须以创新、协调、绿色、开放、共享五大发展理念为根本遵循，下大力气破解制约全面建成小康社会的重点难点问题。坚持创新发展，就是要围绕解决发展动力问题，将创新作为引领发展的第一动力，大力推进以科技创新为核心的全面创新。坚持协调发展，就是要围绕解决发展不平衡问题，统筹处理城乡发展、区域发展、经济社会发展等方面关系，不断增强发展的协调性和整体性。坚持绿色发展，就是要围绕解决人与自然和谐问题，坚定走生产发展、生活富裕、生态良好的生态文明发展路子，增强永续发展能力。坚持开放发展，就是要围绕解决内外联动问题，发展更高层次的开放型经济，以扩大开放带动创新、推动改革、促进发展。坚持共享发展，就是要围绕解决社会公平正义问题，使全体人民在共建共享发展中有更多获得感，朝着共同富裕方向稳步前进。

关于发展目标。按照市委确定的“美丽雅安、生态强市”战略定位，《规划纲要》提出了“十三五”期间的具体发展目标建议是：保持高于全国、全省的经济增长速度，质量和效益得到明显提升；人民生活水平和质量明显提高；公民素质和社会文明程度普遍提升；生态文明建设取得显著成效；重要领域和关键环节改革实现重大突破。按照规划，到“十三五”末，我市地区生产总值争取再上一个台阶，力争达到1000亿元；产业结构、动力结构实现优化升级，产业迈向中高端水平，经济迈入创新驱动、内生增长轨道；全面消除绝对贫困，贫困村全部摘帽，城乡居民收入保持较快增长，收入差距明显缩小，基本公共服务均等化水平稳步提高；市场在资源配置中的决定性作用充分发挥，开放型经济新机制基本形成，法治政府建设进一步加强，各项事业步入法治化轨道；“美丽雅安、生态强市”建设取得新成效，生态环境受到严格保护，生态优势得到有效转化，生态经济实现快速发展，生态红利持续富民强市，生态家园魅力充分彰显，生态文明能够全国示范，走出一条“让绿水青山变金山银山”的特色发展之路。

关于发展重点。《规划纲要》明确提出，在“十三五”期间，我市将以践行重建新路为中心，以建设“美丽雅安、生态强市”为总揽，以打“硬仗”、补“欠账”为主线，以转化优势、特色发展为路径，决战重建脱贫“双攻坚”，大力实施工业强市、生态富民、交通会战、城乡提升“四大行动计划”，突出项目、开放、园区、企业“四大工作抓手”，统筹推进经济建设、政治建设、文化建设、社会建设和生态文明建设，确保与全国、全省同步全面建成小康社会。决战重建脱贫“双攻坚”是雅安同步实现全面小康的重大任务，必须以高度的政治责任感和历史使命感，强化思想自觉、行动自觉，咬定目标、苦干实干，坚决打赢这两场硬仗。“四大行动计划”“四大工作抓手”是雅安贯彻落实中央“五大发展理念”的切入点和具体化，对建设“美丽雅安、生态强市”具有战略性、全局性的牵动作用，必须大力实施，全面落实，见到实效。

2016年工作安排

2016年是灾后重建“三年基本完成”的收官之年，也是“十三五”决胜全面小康的开局之年。做好今年工作，意义十分重大。

今年政府工作的总体要求是：认真贯彻落实党中央、国务院，省委、省政府和市委的决策部署，牢固树立并贯彻“五大发展理念”，主动适应把握引领经济发展新常态，以建设“美丽雅安、生态强市”为总揽，决战重建脱贫“双攻坚”，大力实施“四大行动计划”，突出“四大工作抓手”，全面完成灾后恢复重建规划目标任务，努力保持经济平稳健康发展和社会大局和谐稳定，为“十三五”发展开好局、起好步。

今年经济社会发展主要预期目标建议：地区生产总值增长8%以上；完成全社会固定资产投资550亿元左右；规模以上工业增加值增长9%；社会消费品零售总额增长10%；地方一般公共预算收入增长7.5%；城镇居民人均可支配收入增长8%，农村居民人均可支配收入增长9%；城镇登记失业率控制在4%以内；城镇化率提高1.2个百分点以上；完成省定节能减排和环保目标任务。

为实现上述目标，我们将重点抓好以下工作。

一、决战重建攻坚，奋力夺取灾后恢复重建的全面胜利

坚持重建中心不动摇，既立足当前全面完成重建规划任务，又着眼长远加快灾区发展振兴，向党和人民交一份满意的答卷。

全面完成重建任务。坚持时间倒排、任务倒逼，在保障质量安全的前提下加快推进项目建设，确保到4月20日重建规划项目完工率、投资完成率均达95%；到7月20日全面完成重建规划任务。严格按程序做好重建项目竣工验收、结算（决算）审计、资产移交、产权登记、档案整理等工作，建立健全重建成果管护运营机制，把各类重建成果管理好、使用好、维护好。

整体提升重建水平。按照省重建委关于“展现灾区浴火重生后的新面貌、新城镇、新产业、新生活”的要求，继续抓好重建水平整体提升。做好城乡住房重建配套基础设施建设，建立健全服务管理机制，提升群众居住品质。加强公共服务设施后续能力建设和运行管理，加大专业技术人才引进和培训力度，充分发挥好公共服务设施项目的效益。继续推进中心城区、6个县城和16个重点镇重建项目建设，突出抓好“一城四镇”的管理提升、品位提升，积极培育新业态，展示新形象。切实抓好“两道一路”等重要交通干线沿线风貌管控，推动重建项目周边环境整体提升。强化灾区新形象的宣传，形成一批展示重建成果和特色的新村、产业、文化等标志性品牌。系统总结灾后重建新路子的雅安实践，为中央和省级层面完善治理体系、提升治理能力做好基础性工作。

大力实施产业重建。坚持恢复重建与发展振兴相结合，以工业园区建设为载体抓好工业重建，进一步提升园区承载能力，创新园区管理体制机制，促进工业集约集聚发展；以发展现代农业为抓手加快农业重建，强化产业基地和农业园区建设，全面提升3条百公里百万亩特色产业走廊建设水平；以生态文化旅游融合发展为重点推进服务业重建，一手抓旅游景点打造，一手抓乡村旅游发展，催生一批旅游特色乡镇和旅游精品村寨。继续深化和拓展与7个援建市的产业合作，主动强化与9个省级部门的沟通对接，确保7月20日前助雅招商项目开工建设50个以上。

牢牢坚守工作底线。将坚守“六位一体”工作底线贯穿重建始终，持续开展“回头看”和专项检查工作，强化对项目安全、质量、资金使用等环节的全程监管，确保科学重建、阳光重建、廉洁重建。

二、决战脱贫攻坚，为同步实现全面小康打下坚实基础

全面落实《四川省农村扶贫开发条例》，严格按照“六个精准”要求，扎实开展“五个一批”行动计划，大力实施“3+13”政策组合，实现减贫1.2万人、贫困村“摘帽”150个。

突出精准推进脱贫攻坚。扎实开展建档立卡“回头看”，确保扶持对象精准。科学编制扶贫项目规划，确保项目安排精准。健全扶贫资金管理审核机制，提高资金使用效益，确保资金使用精准。制定贫困村脱贫规划和贫困户脱贫计划，落实到村到户帮扶措施，确保措施到户精准。落实“五个一”帮扶机制〔注25〕，做到沉得下、干得实、有成效，确保因村派人精准。建立科学合理的脱贫验收评估机制和动态统计监测机制，确保脱贫成效精准。

把握重点推进脱贫攻坚。完成“五个一批”行动计划归类认定，编制《“五个一批”脱贫攻坚行动计划实施方案》。突出灾后重建帮扶一批，抓好基础设施提升和产业重建发展，探索灾区重建和脱贫攻坚互融互促的新路子。坚持扶持生产和就业发展一批，建立产业园区、企业吸纳农村贫困群众就业激励机制，引导有劳动能力的人员积极发展生态富民产业，实现就业增收。坚持生态扶贫和移民搬迁安置一批，探索建立生态扶贫机制，将搬迁安置工作与新型城镇化和新农村建设有效衔接。坚持低保政策兜底一批，实现低保线和扶贫线“两线合一”。坚持医疗救助扶持一批，实施健康扶贫工程，将贫困人口全部纳入重特大疾病救助范围。

突破难点推进脱贫攻坚。针对脱贫攻坚的重点区域、重点领域、重点环节，细化落实13个专项方案，突出抓好地震灾区、民族地区和水电库区扶贫，深入推进九项重点扶贫工作，切实解决贫困群众住房难、行路难、饮水难、用电难、通信难、上学难、就医难、创业难、生活困难和文化活动不方便等问题。

创新机制推进脱贫攻坚。建立健全财政扶贫投入稳定增长机制，加大专项扶贫资金、涉农资金、社会组织非定向帮扶资金等整合力度，保障扶贫重点项目投入。完善社会力量参与机制，引导更多金融资金和社会资本参与扶贫开发。强化脱贫攻坚责任机制，全面落实扶贫目标、任务、资金、权责“四到县”制度。

三、强化项目投资拉动，着力保持经济稳定增长

突出把握项目抓手，扎实开展“项目年”工作，充分发挥项目投资对稳定经济增长的关键作用。

强化项目储备申报。主动对接国家重大工程包、“十三五”规划、政府投资项目三年滚动投资计划，策划、包装、储备一批管全局、利长远、带动性强的重大项目，重点编制一批基础设施建设项目、民生领域项目和重点产业招商项目，确保储备项目投资动态保持在3000亿元以上。积极加强对上汇报沟通，争取更多项目纳入国家和省上“盘子”，形成“科学谋划一批、包装储备一批、争取引进一批、开工建设一批、竣工投产一批”的滚动发展局面。

加大项目推进力度。落实市级领导协调推进重大项目制度，实施县（区）、部门项目工作“一把手”负责制，进一步建立健全考核奖惩机制。科学制定实施项目投资计划，全力推进重大项目开工和建设，实施市以上重大项目430个，完成投资312.6亿元，其中新开工项目137个，完成投资85.6亿元。创新项目投融资机制，发挥政府投融资平台作用，切实解决好项目资金缺口问题。积极搞好协调服务，在项目审批、供地、用电、交通等方面予以重点保障，确保项目建设顺利推进。

优化项目投资结构。提高产业投资比重。突出抓好工业投资，确保完成工业投资166亿元以上。积极推进服务业重大项目实施，不断扩大服务业投资规模。紧扣生态文化旅游融合发展试验区建设，加大对农旅融合产业的投资引导。积极鼓励房地产市场投资和消费，促进房地产行业健康发展。进一步激发民间投资活力，继续推出和实施一批政府与社会资本合作（PPP）项目，吸引更多民间资本参与重点项目建设。

四、大力实施工业强市行动计划，加快推进新型工业化进程

坚持走新型工业化道路，突出把握园区和企业抓手，推动工业经济总量扩张、结构优化、质量提升。

加快提升园区发展水平。坚持适度集中、形成规模、体现特色的原则，进一步优化工业园区规划布局。强化水、电、路、气、标准厂房等园区基础设施和政务服务、投融资担保等公共服务平台建设，提高园区综合承载能力，建立健全管理服务“一体化”模式。推进芦天宝飞地产业园区（雅安经开区）创建国家级经济技术开发区工作，挖掘芦山产业集中区、汉源工业园区、石棉工业园区、荥经循环经济示范园区、成雅工业园区以及天全、宝兴工业发展集中区的优势和潜力，不断提升园区规模和效益。力争芦天宝飞地产业园区（雅安经开区）营业收入突破200亿元，全市工业园区营业收入突破500亿元。

培育壮大骨干特色产业。一手抓骨干产业优化提升，一手抓特色产业做大做强，通过建链、补链、强链，着力壮大清洁能源、新材料、机械装备制造、农产品加工四大骨干产业；通过引进、培育、孵化，大力发展新能源汽车、电子信息、生物医药、饮料食品、碲铋合金及晶体材料和精细化工六大特色产业，确保四大骨干产业、六大特色产业产值分别达到510亿元和170亿元。加快重点工业项目建设，确保中恒天汽车、格纳斯二期等在建项目建成投产，新筑通工新能源公交汽车、建安车桥等建成项目量产达效。

大力支持企业做大做强。坚持抓大带小，分类推进骨干龙头企业、优势成长型企业和中小企业发展。实施龙头企业培育计划和中小企业升规计划，鼓励企业通过强强联合、兼并重组和投资合作，进一步壮大规模、提升效益，新增规模以上企业20户以上。整合扩大政府技改支持资金，组织实施三九、雅化、金红叶、一名微晶等50个重点技改项目，完成技改投资65亿元以上。充分发挥行业协会、商会的作用，切实关爱民营企业家成长。

五、大力实施生态富民行动计划，切实转化生态资源优势

牢固树立绿色发展理念，加快把生态资源优势转化为产业优势、经济优势和发展优势，推动生态红利持续富民强市。

持续夯实绿色生态本底。将生态文明理念融入发展全过程，持续推进国家生态文明先行示范区建设。加强重要生态功能区和生态系统建设，严格划定和控制生态保护红线，积极探索碳排放权交易。启动国家级园林城市创建工作，大力实施天然林保护、新一轮退耕还林、水土保持、石漠化综合治理等重点工程，加强自然保护区、森林公园、湿地资源和濒危野生动植物保护，森林覆盖率达到63.6%。强化大气污染联防联控，促进大气质量持续改善。实行最严格的水资源管理制度，抓好水污染治理和饮用水源保护。加强环境监测和执法监管，依法严惩环境违法行为。

大力推进生态农业富民。着眼农业增效、农民增收，深化全国休闲农业与乡村旅游示范市建设。围绕3条百公里百万亩特色产业走廊建设，培育壮大“五雅”特色优势产业，新发展产业基地15万亩。强化农产品质量安全溯源体系建设，新增绿色（有机）认证面积13.5万亩，建成国家绿色（有机）农业示范区。加快雅安国家农业科技园区、芦山现代生态农业园、天全南天现代农业科技示范园和汉源现代农业园区建设，规划建设雨城沿周公河冷水鱼产业园区、名山国家茶叶公园、荥经天麻产业园区、石棉黄果柑产业园区、宝兴中药材产业园区，促进农产品初加工、精深加工及综合利用加工协调发展，力争在引进林竹精深加工龙头企业上实现突破。强化农业品牌建设，支持蒙顶山茶、雅安藏茶等品牌做大做强。培育省级产业化龙头企业2户以上，规范发展农民专合组织100家，实现产业化龙头企业销售收入增长8%以上。

大力推进生态旅游富民。树立“生态雅安·全域旅游”理念，加快国家生态文化旅游融合发展试验区建设，全力打造中国国际特色旅游目的地。加快推进碧峰峡、蒙顶山创建国家5A级旅游景区工作，支持飞仙关、邓池沟、汉姜古城、多营藏茶村等创建国家4A级旅游景区，推动牛背山、龙苍沟、汉源湖、百丈湖、王岗坪、轿顶山、周公山、光头山、达瓦更扎等旅游项目开发，完善旅游配套设施建设。着力发展城市旅游，建设独具魅力的旅游城市。大力发展乡村旅游，带动农村群众就近增收。全力争取建设中国雅安大熊猫国家公园，积极申办中国雅安大熊猫国际文化旅游节，打造大熊猫文化旅游走廊和大熊猫生态精品旅游线路。加强与周边地区的旅游协作和线路对接，主动融入“大峨眉”“大香格里拉”“大九寨”等区域旅游大环线，持续提升“熊猫家源·世界茶源”的影响力和美誉度。精心举办旅游节庆活动，着力提高旅游营销实效。实现旅游综合收入增长16%以上，带动直接就业7000人以上。

大力推进生态康养富民，统筹加快现代服务业发展。高标准规划发展康养产业，加快建设国家健康城市，积极争取列入国家医养结合试点市。策划、包装、推出一批康养项目，重点打造温泉康养、森林康养和阳光康养旅游度假区。支持社会力量兴办医养结合机构，鼓励养老机构与医疗卫生机构开展多种形式的合作。在新村聚居点开展休闲度假养老服务项目建设，拓宽农民增收渠道。大力发展现代物流业，加快建设“一园区、三中心、四服务站”〔注26〕现代物流体系。大力发展现代金融业，推进金融产品服务创新。大力发展电子商务，加快农村电子商务服务网点建设。大力拓展消费领域，积极培育新的消费热点。

六、大力实施交通会战行动计划，加快推进重点基础设施建设

坚持交通先行，统筹加快水利、能源、农村、信息等基础设施建设，不断改善发展条件。

加快对外通道建设步伐。围绕建设川西综合交通枢纽，推动雅安与全省五大经济区〔注27〕及云南、西藏相联系的战略通道建设，有效链接“一带一路”国际运输大通道和长江经济带综合立体交通走廊。加快推进川藏铁路成都（朝阳湖）至雅安段建设，力争启动川藏铁路雅安至康定段建设。加快推进雅康高速公路建设，全面启动峨汉高速公路和成雅快速通道建设。扎实做好雅眉乐铁路、成雅高速公路复线、泸石高速公路等重点项目前期工作。

提升市内路网建设水平。全面完成国道318线改造，实现国道351线主体工程完工。启动国道351线对岩至多营连接线、省道105线雅安至上里段、省道433线泗坪经三合至泸定段建设，全面完成芦山至龙门公路、宝兴经永富至河口大桥公路建设。全力做好国道318线名山及雅安主城区过境路段、国道549线石棉段、省道217线石棉至甘洛段、省道308线天全经荥经至瓦屋山段改建等项目前期工作。进一步改善农村出行条件，提高村组和重点林区路网覆盖率，促进跨行政村道路的互联互通。

统筹推进重点基础设施建设。加快重点水利工程建设，推进铜头引水、九龙水库、南郊水厂引水工程建设，全面完成永定桥水利工程主体工程建设。加快能源基础设施建设，力争启动老鹰岩水电站建设，加快锅浪跷水电站、民治水电站、出居沟水电站和110千伏输变电工程建设，大力实施农网改造升级。加快农村基础设施建设，新建高标准农田示范区1.42万亩，解决3.5万人安全饮水问题。推进“智慧雅安”“光网雅安”“无线雅安”建设，支持通信、广电运营商实施信息基础设施演进升级工程。

七、大力实施城乡提升行动计划，加快推进新型城镇化进程

按照中央“五大统筹”〔注28〕要求，科学完善城乡规划体系，同步加强建设管理，着力提高城镇化水平，促进城乡大提升、大发展。

科学完善城乡规划。统筹生产、生活、生态三大布局，围绕宜居宜游宜商宜业做好全域规划。加快城市特色规划研究，完成旅游城市、历史文化名城保护、城区地下综合管廊以及商业业态、专业市场等规划编制，全面开展城市设计。完成芦山、宝兴、天全县城总规修编，全面完成全市镇（乡）规划编制工作。

统筹加快城乡建设。以“一城三廊六片”〔注29〕为重点，加快中心城区扩容提质。完善姚桥新城功能配套，启动火车站站前广场建设，着力推进雅安万达商业广场、国盛·雅安国际商贸城等城市综合体建设。加快大兴新区建设，开工建设南外环线和大兴核心区生态系统工程，建成大兴污水处理厂一期工程及配套管网。加快熊猫绿岛开发，开工建设水中坝桥梁工程和熊猫绿岛公园。加快老城片区城市棚户区改造，统筹做好地下管网建设，试点开展既有住宅电梯增设工作，完成康藏路、挺进路改造和陇西河桥梁及连接道路建设。完善城市绿地系统，实施大兴河道及湿地综合整治、青衣江两岸绿化亮化等项目。加快雅安生活垃圾焚烧发电厂建设，基本建成城区生活垃圾压缩站。加快雨城区、名山区、芦天宝飞地产业园区（雅安经开区）同城化发展，有序推进多营、北郊、对岩片区建设。着力增强6个县城综合承载能力，全面完成“百镇建设行动”7个试点镇建设任务，新建幸福美丽新村200个。

大力加强城乡管理。以“两城”创建为契机，稳步推进城市管理领域大部门制改革，充分发挥街道办和社区的基础作用，广泛发动群众参与城市管理。深入推进城乡环境综合治理，大力开展城市管理违法建筑、卫生秩序、交通秩序、经营秩序、扬尘污染五大专项治理行动，坚决查处纠正私搭乱建和城市“五乱”行为。加大市政基础设施建设力度，完成魏家岗、西城等7个城区农贸市场标准化建设，新建立体停车位1000个，新建和改造地面诱导停车位1700个。优化城市路网，治理易堵路段，提高城区通行能力。实施“千村整治”工程，促进农村环境秩序进一步好转。

提高户籍人口城镇化率。坚持政府投入和市场融资相结合，鼓励社会资本参与新型城镇化建设。有序推进撤乡建（并）镇和乡（镇）改街道办、村改居工作，加快推进农业转移人口市民化。继续深化户籍制度改革，全面实施居住证制度，着力提高户籍人口城镇化率。

八、坚持改革创新，着力培育经济发展新动能

大力实施创新驱动发展战略，深入推进重点领域各项改革，进一步增强发展动力，激发发展活力。

坚持创新驱动发展。大力培育高新技术企业和科技型中小微企业，新增高新技术企业3户、科技型中小微企业20户，实现高新技术产业产值80亿元以上。积极支持国家和省级工程技术研究中心、重点实验室等科研平台建设，着力构建政、产、学、研、用相结合的技术创新体系。加快推进科技成果转化，实施市级以上重大科技成果转化项目30个。支持国家茶检中心和雅安现代畜禽创新示范园区建设，推进碲铋产业技术研究院组建工作。强化体制机制创新，推动军民深度融合发展。推动大众创业、万众创新，加快省级孵化器建设，建成各类孵化载体10家以上；建立健全返乡创业信息沟通和项目对接机制，支持在外雅商和农民工、农民企业家返乡创业；实施大学生就业促进计划和创业引领计划，加快推进大学生创新创业园和创新创业俱乐部建设。

着力深化各项改革。继续深化农村产权制度改革，按期完成土地承包经营权确权颁证工作，全面开展农村房屋不动产权属证书颁证工作，加快建立县（区）农村产权流转平台。深化国资国企改革，加快实现从管资产向管资本转变，促进国有资本布局的动态优化和调整。深化财税体制改革，实行全口径预算管理。深化金融领域改革，支持雅安农村商业银行深化改革，继续做好雅安市商业银行增资扩股工作；拓宽直接融资渠道，推动具备条件的企业挂牌上市融资。有序推进资源要素配置市场化改革，持续推进医药卫生体制、教育领域和文化体制等改革。

扎实抓好结构性改革重点任务。围绕“去产能、去库存、去杠杆、降成本、补短板”，加强供给侧结构性改革。落实中央减税降费政策，开展降成本增效益专项行动，切实减轻企业负担。科学确定化解过剩产能的目标任务和实现路径，通过兼并重组、消化淘汰、管控增量等方式分类组织实施，淘汰10户企业落后产能。进一步健全担保体系，提高融资担保能力。完善金融监管机制，积极做好处置非法集资和民间理财风险防控工作，坚决守住不发生系统性、区域性金融风险的底线。

九、坚持开放合作，努力提升开放型经济水平

突出把握开放抓手，深化全方位区域合作，努力在招大引强上实现新突破，着力提升开放型经济发展水平。

着力深化区域合作。精准对接国家西向、南向开放和扩大内陆开放的战略布局，积极参与“一带一路”和长江经济带建设。主动融入“1+7”同城化发展，着力推进攀西战略资源创新开发试验区建设，继续深化与成都、乐山、眉山、甘孜、阿坝、凉山等市（州）和相关省级部门、企业的战略合作，共同谋划实施一批重大项目，借势借力推动雅安发展。

着力强化招商引资。大力开展驻点招商、以商招商、中介招商、“互联网+招商”，积极组织参加第十六届西博会、首届全球川商返乡发展大会、中外知名企业四川行等重大投资促进活动，精心办好第三届攀西战略资源创新开发试验区投资大会。将招大引强工作纳入目标考核，建立重大招商活动签约项目信息库，严肃查处损害投资环境的行为。引进到位市外资金350亿元以上，引进投资5亿元以上项目10个、10亿元以上项目5个。实现进出口总额4200万美元以上。

着力发展县域经济。坚持“三大片区”〔注30〕整体联动、错位发展，支持县（区）转化比较优势，加快建设现代农业、工业、服务业和生态经济强县。全面落实扩权强县和支持县域经济发展的各项政策，在产业布局、项目争取和政策支持等方面加大帮助、指导、服务力度，充分激发县域经济发展活力。

十、坚持共建共享，努力增强人民群众的获得感

把保障和改善民生作为工作的出发点和落脚点，守住底线、突出重点、完善制度、促进和谐，使发展成果更好地惠及全市人民。

用心办好民生实事。继续加大民生投入，重点实施好“十项民生工程”和“20件民生实事”。坚持以创业带动就业，争创省级创业型城市，城镇新增就业1.6万人。完善社会保险制度，加快推进养老服务体系建设，稳定和扩大城乡居民基本养老保险、城镇职工养老保险覆盖面。健全全民医保体系，推进建立统一的城乡居民基本医疗保险制度。统筹推进社会救助，提高城乡低保水平。开工建设保障性住房和实施危旧房棚户区改造3821套。积极争取国家和省上政策扶持，加快革命老区、民族地区发展。持续抓好水电库区后续建设和发展。

全面发展社会事业。大力发展公益性、普惠性幼儿园，扩充学前教育资源；推进义务教育优质均衡发展，提高普通高中教育质量，加强教师队伍业务能力和师德师风建设；推动职业教育教学改革，支持发展民办教育。落实全面两孩政策。推进城市公立医院综合改革全省试点市建设，组建市妇女儿童中心医院，加快建设雅安川西区域医疗中心；巩固完善分级诊疗制度，引导优质医疗资源下沉。大力实施文化惠民工程，加强文化遗产保护传承，强化文化市场监管，推进广播电视电影基础设施建设；积极开展全民健身活动，举办第三届全市运动会和第四届全市老年人运动会。扎实开展妇女、儿童、老龄、残疾人、社会福利、慈善、红十字工作，切实做好防震减灾、气象、档案、保密、科协、地方志、社会科学、外事、侨务和对台等工作。

深入推进依法治理。全面启动“七五”普法，稳步推进地方立法工作，深入开展“法律八进”〔注31〕活动。坚持预防为主，注重源头治理，强化应急防范与处置，有效化解各类矛盾问题。健全基层依法治理体系，支持群团组织参与社会治理和维护社会稳定。持续强化感恩教育，注重发挥市民公约、乡规民约的积极作用。加强立体化社会治安防控体系建设，强化反恐防暴和反分裂斗争，严厉打击违法犯罪。依法规范信访秩序，大力推行“阳光信访”“法治信访”“责任信访”。抓好危爆物品、道路交通、消防火灾等安全监管，持续深化安全专项整治，坚决遏制重特大安全生产事故发生。实施最严格的食品药品安全监管，从严查处违法违规行为。加强国防动员和民兵预备役建设，做好双拥共建、优抚安置等工作。认真贯彻党的民族宗教政策，加强和改进民族宗教工作。

各位代表，我们深深知道，人民群众对政府工作寄予厚望。今年，我们将聚焦依法行政、转变职能、提高效能，进一步深化法治政府、服务政府和廉洁政府建设。

我们将始终坚持依法行政。依法全面履行政府职责。自觉接受市人大及其常委会的法律监督、工作监督和市政协的民主监督，认真执行市人大及其常委会的决议、决定，认真办理人大代表建议意见和政协委员提案。认真听取民主党派、工商联、无党派人士和人民团体的意见建议，开放接受人民群众和新闻舆论监督。健全依法决策机制，坚持重大决策公众参与、专家论证、风险评估、合法性审查、集体讨论决定制度。强化行政执法规范化建设，提升执法人员法律素质和能力。

我们将始终坚持简政放权。科学处理政府与市场、政府与社会的关系，加快转变政府职能。深化行政审批制度改革，优化完善市、县（区）两级权力清单，编制公布市、县（区）两级责任清单。全面实施“先照后证”制度，推进“事前审批为主”向“事中事后监管为主”转变。大力推广“互联网+政务服务”，加快政务服务中心标准化和电子政务大厅建设，完成全市公共资源交易平台整合。依法规范政务公开，提高政府工作的透明度和公信力。

我们将始终坚持从严治政。以纯政风、严政纪为重点，深入践行“三严三实”，持之以恒落实中央“八项规定”精神，严格执行省委省政府“十项规定”，驰而不息纠正“四风”。加强政务督查和效能监察，坚决纠正不作为、乱作为，坚决克服懒政、怠政，坚决惩处失职、渎职。严格落实党风廉政建设责任制，把廉洁从政贯彻到政府工作各个环节。加强行政监察和审计监督，加大违纪违法案件查处力度，以反腐倡廉的实际成效取信于民。

各位代表，雅安发展已经站在新的历史起点上。让我们紧密团结在以习近平同志为总书记的党中央周围，在省委、省政府和市委的坚强领导下，进一步凝心聚力、拼搏实干，为同步实现全面小康，加快建设“美丽雅安、生态强市”而努力奋斗！
